# Non-type 2 airway inflammation in severe asthma: from bench to bedside

**DOI:** 10.3389/fimmu.2026.1930520

**Published:** 2026-08-24

**Authors:** Hiroki Tashiro, Yuki Kuwahara, Yuki Kurihara, Koichiro Takahashi

**Affiliations:** Division of Hematology, Respiratory Medicine and Oncology, Department of Internal Medicine, Faculty of Medicine, Saga University, Saga, Japan

**Keywords:** asthma, IL-17A, neutrophil, non-type 2 inflammation, obesity

## Abstract

Severe asthma remains a major clinical challenge despite substantial advances in biologic therapies targeting type 2 inflammation. Whereas type 2-high asthma is increasingly well characterized and effectively treated, a considerable proportion of patients exhibit type 2-low and non-type 2 inflammatory phenotypes, which are frequently associated with neutrophilic airway inflammation, corticosteroid insensitivity, poor symptom control, and persistent disease burden. However, the biological mechanisms underlying these phenotypes remain incompletely understood, and effective targeted therapies are limited. In this review, current evidence regarding the clinical characteristics, pathogenic mechanisms, and emerging therapeutic targets associated with non-type 2 airway inflammation including non-allergic, non-eosinophilic and type 2-low asthma is summarized. Clinical studies indicate that obesity, exposure to air pollutants, cigarette smoking, and respiratory viral infections are important contributors to these inflammatory phenotypes and are often associated with increased disease severity and reduced responsiveness to corticosteroids. Mechanistically, activation of innate and adaptive immune pathways involving interleukin (IL)-17A, IL-6, thymic stromal lymphopoietin (TSLP), and osteopontin promotes neutrophilic airway inflammation and airway hyperresponsiveness. Recent findings also highlight the importance of the gut-lung axis, suggesting that alterations in the gut microbiome and its metabolites contribute to systemic and airway inflammation, particularly in obesity-associated asthma. Although several candidate therapeutic targets have emerged, including IL-17A, TSLP, osteopontin, IL-6, and microbiome-related pathways, clinical translation has been challenging, and reliable biomarkers for patient stratification remain lacking. The substantial biological heterogeneity of non-type 2 inflammatory phenotypes further complicate disease classification and therapeutic development. A better understanding of the interactions among environmental exposures, metabolic factors, and immune responses will be essential for refining asthma endotypes and advancing precision medicine approaches for patients with severe asthma characterized by non-type 2 airway inflammation.

## Introduction

Severe asthma is characterized by frequent exacerbation, augmented symptoms related to narrowing of airways, and decreased pulmonary function, which increases use of systemic corticosteroids (1, 2). In the entire population of patients with asthma, the prognosis is worse in patients with severe asthma than in others (3), which contributes to the excess medical costs of approximately 60% (4). Recent molecular analyses related to the severity mechanisms clarified that identification of inflammatory phenotypes including type 2 inflammation and type 2-low or non-type 2 inflammation is needed (5). Specifically, in type 2 inflammation, eosinophils accumulate in airways with elevations of interleukin (IL)-4, IL-5, and IL-13, called type 2 cytokines, involving other immunocompetent cells of antigen-presenting cells (APCs), inflammatory cells, and effector cells such as dendritic cells (DCs), macrophages, B cells, T helper 2 (Th2) lymphocytes, group 2 innate lymphoid cells (ILC2s), mast cells, and basophils (6–8). In addition, the airway epithelium plays a central role in asthma pathogenesis related to type 2 airway inflammation. Typically, in response to airway epithelial damage, cytokines such as thymic stromal lymphopoietin (TSLP) and IL-33 are released and contribute to airway inflammation, making them promising therapeutic targets for patients with severe uncontrolled asthma (9, 10). However, in type 2-low or non-type 2 inflammation, neutrophils predominate, and typically fewer eosinophils migrate into airway epithelial tissue with elevations of IL-17A, IL-1β, and tumor necrosis factor-α (TNFα), called non-type 2 cytokines, involving DCs, macrophages, B cells, group 3 innate lymphoid cells (ILC3s), and type 17 helper T (Th 17) lymphocytes (11, 12). Notably, the definition of type 2-low and non-type 2 inflammation is still controversial, because the phenotype of type 2-low is considered to involve absence of type 2-high biomarkers such as elevated blood eosinophils and fractional exhaled nitric oxide (FeNO), leading to the dominance of neutrophilic or pauci-granulocytic inflammation (13, 14). Furthermore, the phenotype of non-type 2 inflammation includes the dominance of Th1/Th17 immunological responses, which also consequently polarizes neutrophilic airway inflammation (11). Additionally, corticosteroid treatment might suppress the type 2 biomarkers and lead to apparent type 2-low classification (15). Hence, clearly distinguishing between type 2-low and non-type 2 inflammatory phenotypes might be challenging. In this review, the term “non-type 2 inflammation” is used to refer to inflammatory phenotypes associated with non-allergic, non-eosinophilic, and type 2-low asthma, based on the classification proposed by Liu et al. (11). Clinically, several biologics targeting immunoglobulin E (IgE), IL-4, IL-5, IL-13, and TSLP are currently available to treat patients with severe asthma, which contribute to improving the clinical remission rates, including reduction of the systemic steroid dose, exacerbation rate, and asthma-related symptoms, and improvement of pulmonary function with reduction of mucus plugs (5, 16–18). However, these biologics show greater effectiveness for the severe asthmatic patients with type 2 airway inflammation than those with non-type 2 airway inflammation. According to these, understanding the clinical features, the mechanistic basis, and the therapeutic targets in patients with severe asthma characterized by non-type 2 airway inflammatory phenotype is increasingly required. In this review, non-type 2 phenotypes of asthma, as characterized predominantly by neutrophilic and/or IL-17-associated inflammation, are addressed, considering the heterogeneity and overlap of biological mechanisms of the clinical features of non-type 2 inflammatory phenotypes. Comprehensive approaches from bench to bedside are performed to evaluate the detailed mechanisms of severe asthma patients with non-type 2 airway inflammation. Better understanding of this form of asthma will contribute greatly to the development of therapies for patients with severe asthma.

## Clinical characteristics of patients with asthma featuring non-type 2 inflammation

### Possible clinical characteristics of non-type 2 inflammatory phenotypes in severe asthma: cluster analysis and stratified analysis

Due to accumulated clinical data calculated by cluster analysis and a comparison based on stratified analysis, clinical phenotypes focusing especially on inflammatory features have tended to be clarified (19). In the well-known cluster analysis by Wenzel, the pathophysiology of asthmatic patients can be broadly divided into type 2-high and non-type 2 phenotypes. Type 2-high asthma ranges from typically mild early-onset allergic asthma to more severe late-onset eosinophilic disease, whereas exercise-induced asthma is generally a milder type 2-high subtype. Non-type 2 asthma includes obesity-related, smoking-associated, neutrophilic, and low-inflammatory forms of the disease. The prevalence and severity vary among these phenotypes, with late-onset eosinophilic asthma often being associated with greater severity (20). In addition, recent data indicated that, compared with severe asthma patients with type 2-high inflammation, patients with non-type 2 inflammation show different clinical features, especially with respect to severity, steroid responsivity, and clinical phenotypes, and the factors inducing polarization to non-type 2 inflammation, even though the specific biomarkers reflecting the phenotype of non-type 2 inflammation are still not established (11, 12). A population-based study of 896 patients with adult asthma reported that patients with low levels of type 2 markers having less than 0.15 × 10 ^9^/L of blood eosinophils, 20 parts per billion (ppb) of FeNO, and no history of allergen-driven disease showed frequent emergency room visits related to asthma compared with patients with high levels of type 2 markers (21). Cluster analysis of a Japanese population with severe asthma in a research program clarified that, in the 5 clusters, patients with non-type 2 inflammation and a relatively high body mass index (BMI) showed the worst asthma control, with frequent emergency room visits, a higher ratio of patients with treatment based on the Strategy of Global Initiative for Asthma (GINA) step 5, and not well controlled by treatments compared with other clusters (22). Indeed, eosinophils and neutrophils, which are the major inflammatory cells in type 2-high and non-type 2 inflammation, respectively, have different biological responses to glucocorticoids (23). Specifically, glucocorticoids directly induce apoptosis of eosinophils, but they inhibit apoptosis of neutrophils (24), consistent with the clinical endotypes of steroid resistance mentioned above. According to these data, the severity of asthmatic patients reflected by their inflammatory phenotype, whether type 2-high or non-type 2 inflammation, differed, supported by the biological mechanisms of their principal inflammatory cells of eosinophils and neutrophils, but more precise basic data and clinical research are needed for further understanding.

## Obesity-induced asthma pathophysiology

Obesity is one of the important clinical forms of asthma, and obesity is associated with greater severity of asthma, along with increased incidence of asthma, than in lean individuals (25, 26). A previous cluster analysis indicated that asthmatic patients with obesity characterized by non-eosinophilic inflammation are an important phenotype in the whole population of asthma patients, and these patients are characterized late onset, female predominance, and high symptom expression (27). In addition, obesity is associated with a diminished therapeutic response to corticosteroids in asthma (28). For example, obese patients are less likely to achieve asthma control following inhaled corticosteroid treatment, with or without long-acting β2-agonists (29). This reduced responsiveness to corticosteroids may be partly explained by impaired glucocorticoid-mediated anti-inflammatory signaling, including decreased induction of MAP kinase phosphatase-1 (30). Furthermore, obese patients with severe asthma often exhibit persistent type 2 inflammatory activity despite corticosteroid therapy (31). The limited efficacy of steroids may also be related to the higher prevalence of non-atopic asthma phenotypes in obese individuals, which are less dependent on the allergic pathways targeted by corticosteroids (25, 26). In the Japanese population of asthma patients, compared with patients with a normal BMI (< 25 kg/m^2^), obese individuals with a high BMI (> 25 kg/m^2^) showed a significantly higher rate of annual exacerbations, lower blood eosinophils, and decreased pulmonary function including forced expiratory volume in one second (FEV_1_) and forced vital capacity (FVC), and a higher rate of treatment with high-dose inhaled corticosteroids (ICS) (32). In other real-world data, obesity was significantly correlated with decreased pulmonary function in patients with asthma, but not in those without asthma, which indicated that obesity has a specific effect in patients with asthma (33). In mice, sensitization and exposure to house dust mite (HDM) induces airway hyperresponsiveness (AHR) and airway inflammation with eosinophils and neutrophils accumulating in airways, reflecting the pivotal pathophysiology of asthma (34, 35). In obese mice fed a high-fat diet, greater AHR and neutrophilic airway inflammation with elevation of IL-17A levels in lung were seen compared with lean mice (36, 37). Genetic obese mice, *ob/ob* or *db/db* mice, which lack the gene coding leptin or its receptor, showed greater AHR and neutrophil cell counts in bronchoalveolar lavage fluid (BALF) exposed to ozone, one of the triggers, as an air pollutant than wild-type lean mice (38, 39). Others also reported that allergen challenge by ovalbumin (OVA) increased AHR in genetic obese mice more than in lean mice (40). The mechanisms underlying obesity-induced severe asthma remain controversial because multiple factors, including adipose tissue-derived adipokines, obesity-related comorbidities, systemic inflammation, and alterations in the microbiome, are involved as mentioned later (25). Furthermore, it remains challenging to determine whether type 2-high or non-type 2 inflammation predominates in this heterogeneous population. Collectively, these findings suggest that obesity promotes non-type 2 inflammation in a subset of patients with asthma, although other studies have also suggested that obesity may exacerbate type 2 inflammation in certain individuals with asthma (41).

## Possible external triggers of non-type 2 inflammation in severe asthma

### Air pollutants

Air pollutants such as ozone, particulate matter (PM) 2.5, and nitrogen oxides (NOs) are possible triggers for asthma as external stimulants (42, 43). Importantly, they have the capacity to induce non-type 2 airway inflammation. In a meta-analysis of the impact of PM2.5 on asthma-related emergency department (ED) visits, exposure to ambient PM2.5 has an adverse effect on asthma ED visits even after short-term exposure, and children are a high-risk population when PM2.5 concentrations are increased (44). Others also reported that PM2.5 exposure was positively related to cumulative asthma-related hospital admissions within a 0–7 day lag period, and an increment of 10 µg m^-^³ in the PM2.5 concentration was linked to a 0.49% increase in asthma admissions (45). In mouse or rat models, PM2.5 exposure augmented AHR in a dose-dependent manner in an asthmatic mouse model caused by OVA, and accumulation of neutrophils in airways with elevation of IL-17A levels was observed (46, 47). As mechanisms, exposure of bronchial epithelial cells to PM2.5 induces CXC motif chemokine ligand 8, which contributes to migration of neutrophils to tissue (48) and neutrophil extracellular traps (NETs), which are also involved in PM2.5 inducing neutrophilic inflammation (49). Other pollutants such as nitrogen dioxide (NO_2_) enhance the pathophysiology of asthma (43). For example, in clinically, repeated short-term exposure to ambient-level NO_2_, inflammatory responses to inhaled allergens were enhanced in individuals with allergic asthma. Although symptoms and lung function were not significantly different in this study, the findings suggested that NO_2_ may amplify airway inflammation by increasing the asthma-related pathophysiology after allergen exposure (50). In mice sensitized by OVA, subsequent exposure to NO_2_ at 25 ppm induced neutrophils along with eosinophils in BALF and AHR, which indicated that NO_2_ also has the potential to polarize non-type 2 inflammatory phenotype (51). Notably, NO_2_ is one of the major harmful components of diesel exhaust, and the particle enhances the Th17 response, with augmentation of IL-17A levels in mice and humans, which might be involved in the pathophysiology of NO_2_-induced non-type 2 airway inflammation as the mechanism (52). Ozone, another air pollutant, is also one of the important triggers of asthma in terms of exacerbations (53, 54). A huge observational study of 1.6 million asthma hospitalizations in the United States reported that a 1-ppb increase of ozone concentration was significantly associated with an increased risk of hospitalization related to asthma (55). Importantly, inhalation of ozone in humans induces neutrophil accumulation in airways with AHR, reflecting the non-type 2 inflammatory phenotype (56–58). In mice, ozone exposure at 2 ppm for 3 hours showed AHR and elevation of neutrophils in BALF (59–63). In addition, obesity, which is one of the severe forms of asthma, as mentioned above, induces greater AHR and neutrophilic airway inflammation with elevation of IL-17A levels in BALF in mice (38). Importantly, ozone exposure further increases neutrophil counts in BALF and AHR caused by HDM stimulation (9). These data indicated that ozone exposure has an effect on patients with asthma in terms of polarization to the non-type 2 inflammatory phenotype.

### Cigarette smoke

Cigarette smoke includes various harmful molecules, which affect the pathophysiology of asthma via airway epithelial cells (64). Importantly, cigarette smoke is associated with the incidence of asthma and an increased severity level, supported by clinical and basic evidence (11, 65). Data from the Canadian National Population Health Survey indicated that smoking was associated with a substantially increased prevalence of asthma in women, with female smokers experiencing a 70% greater prevalence than nonsmokers. This interaction between smoking status and sex was the most notable in women under 25 years of age and was more evident in heavy smokers than in light smokers or nonsmokers (66). Others also reported that, in a retrospective, cohort study, smoking was significantly associated with the risk of asthma development, and the risk of new-onset asthma was clearly correlated with the dose of tobacco in individuals with allergic rhinitis (67). Factors related to asthma severity, including frequency of exacerbations, decreased lung function, and corticosteroid insensitivity, are also correlated with the smoking level (68–70). Importantly, exposure to cigarette smoke potentially induces non-type 2 airway inflammation. Boulet et al. compared airway inflammatory phenotypes and lung function between 22 smoking patients with asthma and 27 non-smoking patients with asthma, and the ratio of neutrophils, but not eosinophils, in sputum was significantly higher with lower FEV_1_/FVC in smokers than non-smokers (71). Others also reported that, in biopsy samples of airway tissues, expressions of IL-17A and IL-8 and neutrophil numbers were increased in the bronchial mucosa of asthmatic smokers compared with non-smokers (72). In addition, cells positive for neutrophil elastase and IL-8 were greater in smoking asthmatics than in non-smoking asthmatics (73). Indeed, inhalation exposure of normal mice showed elevated neutrophils in BALF dependent on exposure duration (74). In asthmatic mice sensitized and challenged by HDM, exposure to cigarette smoke extract increased neutrophils in BALF, with upregulation of gene expressions related to the IL-17A signaling pathway and NET formation (75). As important mechanisms, steroids cannot attenuate the increased neutrophils caused by cigarette smoke exposure in asthmatic mice, even though eosinophils are decreased by steroid treatment (76), which supported the evidence of steroid resistance mentioned above. According to these data, cigarette smoke is an independent risk factor for the development of non-type 2 inflammatory phenotypes in patients with asthma.

### Viral infections

There is increasing evidence that respiratory viral infections, especially respiratory syncytial virus (RSV) and human rhinovirus (HRV), are closely related to the development and augmentation of asthma (77, 78). Sigurs et al. reported that, in 140 children who underwent follow-up physical examinations, skin prick tests, and serum IgE tests, the prevalence of asthma was 30%, whereas that of any wheezing was 68% in the RSV-infected groups, significantly higher than the 3% and 34%, respectively, in the control group, supported by similar prospective and retrospective studies of huge numbers of participants (79–82). In terms of HRV, the wheezing illness caused by HRV infection is significantly associated with the development of childhood-onset asthma with genetic variants at the 17q21 locus in huge cohorts of children (83). Others have also reported that, in 259 children who were prospectively followed from birth to 6 years of age, a wheezing illness caused by HRV infection in infancy and early childhood was an important predictor of the development of asthma at the age of 6 years (84), even though these inflammatory phenotypes from childhood might involve polarization to type 2 inflammation characterized by atopic pathogenesis. In a meta-analysis involving adults, the prevalence of asthma was high, at 19.3%, in RSV-infected adults, and the incidence rate ratio of hospitalization following RSV infection was 6.7 - 8.2 in patients with asthma compared with those without RSV infection (85). Venarske et al. prospectively analyzed 101 adults with hospital admissions for an asthma exacerbation, and HRV was detected in 21% over a 4-year observation period (86). In Japan, Kan-O et al. also reported that, in 64 outpatients with adult asthma exacerbations, viral pathogens were identified in 31 patients, and HRV was the most commonly detected virus (87). According to these data, viruses, especially RSV and HRV, are strongly associated with the development and augmentation of asthma. Neutrophils in BALF, with induction of Th17 cells, as well as AHR, are increased by infection with RSV in an asthmatic mice model stimulated by OVA (88). HRV also augments allergen-induced AHR and neutrophilic airway inflammation along with the elevation of type 2 cytokines including IL-4 and IL-13 (89). Poly(I:C) is widely used as a surrogate for viral double-stranded RNA generated during replication of respiratory RNA viruses such as RSV and HRV (90). Asthmatic mice stimulated with poly(I:C) showed AHR and neutrophilic airway inflammation with elevation of IL-17A levels in lung, along with IL-1β, IL-5, IL-13, and CXCL2 level elevations (91). These data indicated that virus infection has the possibility of affecting the development and augmentation of asthma with non-type 2 inflammatory phenotypes, even though the opposite results for augmentation of type 2 inflammation have also been reported (92, 93).

## Mechanistic basis and future possible therapies for non-type 2 airway inflammation in severe asthma

### IL-17A

IL-17A is one of the strong activators of neutrophils, and the sources of IL-17A are considered to be ILC3s and Th17 lymphocytes (94, 95). Previous studies indicated that Th17 lymphocytes are regulated by antigen-presenting cells such as DCs, B cells, and macrophages via the T cell receptor (TCR) – major histocompatibility complex class II (MHC2) pathway (96, 97) and the ox40-ox40L pathway (98, 99). Importantly, IL-17A is involved in the pathophysiology of asthma with neutrophilic airway inflammation, and it might be a candidate future therapeutic target (100). As mentioned previously, obesity is one of the major forms of asthma with non-type 2 airway inflammation. In mice, diet-induced obese mice fed a high-fat diet showed greater AHR with elevated IL-17A levels and accumulation of ILC3s. Notably, *Il17a*-deficient mice showed attenuated AHR compared with wild-type mice, indicating that obesity-associated AHR is facilitated by IL-17A signaling through ILC3s. Furthermore, activation of the NOD-like receptor family pyrin domain-containing (NLRP) 3 inflammasome promoted macrophage-derived IL-1β production, leading to expansion of pulmonary ILC3s and enhanced IL-17A-mediated airway inflammation. Consistently, *Nlrp3*-deficient mice or pharmacological blockade of IL-1 signaling markedly attenuated obesity-induced AHR, suggesting that the NLRP3–ILC3–IL-17A axis plays a pivotal role in the pathogenesis of obesity-associated asthma supported by the animal study (101). We also reported that diet-induced obesity mice fed a high-fat diet showed higher neutrophil counts in BALF with AHR in asthmatic mice stimulated with HDM than lean mice. IL-17A concentrations in lung were also higher in asthmatic mice with obesity than in those without obesity (36, 37). Ozone exposure also increases neutrophils, but not eosinophils, in BALF (58, 60) and genetically induced obese mice, called *db/db* mice, showed further increased neutrophils with elevation of airway IL-17A levels (38, 39). Importantly, IL-23, a molecule that affects the survival and activation of Th17 lymphocytes and IL-17A-expressing CD45-positive cells, considered Th17 lymphocytes, was higher in obese mice with ozone exposure than lean mice, and neutralization of IL-17A by antibody attenuated ozone-induced increases in AHR in *db/db* mice (102). To understand the effect of IL-17A signaling in asthma pathogenesis, Mckinley et al. demonstrated that IL-17A production by Th17 lymphocytes derived in OVA-specific TCR-transgenic mice was refractory to dexamethasone treatment, even though IL-13 production from Th2 lymphocytes was attenuated by the identical dose of dexamethasone, which supports steroid resistance in IL-17A signaling as a severity mechanism in asthma showing in the animal models (103). Although the IL-23/IL-17 axis is mechanistically implicated in non-type 2 asthma, translation of this pathway to the clinic has so far been unsuccessful. In a phase 2a trial, risankizumab, an antibody targeting IL-23p19, failed to improve severe asthma outcomes and was associated with earlier asthma worsening compared with placebo in the human study (104). Similarly, brodalumab, an anti-IL-17 receptor A antibody, did not show significant overall benefits in asthma control, lung function, or symptoms in patients with moderate-to-severe asthma, suggesting that future studies may require more potent pathway modulation and careful enrichment for non-type 2 inflammatory phenotypes in asthmatic participants (105). Notably, IL-17A also induces IL-8, a potent neutrophil chemoattractant, thereby amplifying neutrophil recruitment and activation in the airways (106). In the experimental model, IL-17A induces IL-8 production by airway epithelial cells, and this response is markedly enhanced in the presence of TNF-α. These findings suggest that IL-17A contributes to neutrophilic airway inflammation through enhanced IL-8-mediated neutrophil recruitment (107), supporting the role of the IL-17A–IL-8 signal in neutrophilic airway inflammation.

### TSLP

TSLP is one of the epithelial cell-derived cytokines such as IL-33 and IL-25, and it stimulates ILC2s and DCs to mediate type 2 airway inflammation (5, 108–110). TSLP is generated by epithelial damage as a danger signal and affects asthma pathophysiology, including steroid resistance and airway remodeling (111, 112). In a phase 3 study of tezepelumab, a monoclonal antibody targeting TSLP, the reduction of the annualized rate of asthma exacerbations was greater in patients treated with tezepelumab than in those treated with placebo, and the effect was greater in patients with higher blood eosinophils than in those with lower blood eosinophils, even though the decrease was still significant in patients with lower blood eosinophils of less than 300 cells/μL (113). Indeed, a recent phase 4 study from Japan also indicated that a baseline blood eosinophil count ≥ 300 cells/μL was an independent predictor of clinical remission as an ambitious therapeutic goal in patients with severe asthma (114). In mice, ozone and cold temperature as external stimulants are considered to be TSLP inducers through epithelial damage and exposure to the increased eosinophils in the BALF of the asthmatic mouse model stimulated with HDM and papain, along with increased TSLP levels in the lung (9, 115). Importantly, a single exposure of ozone induces TSLP elevation with increased neutrophils, but not eosinophils, with AHR, and these phenomena were lower in TSLP receptor-deficient mice compared with WT mice. In addition, in single-cell RNA sequencing of lung immune cells, DCs, especially conventional type 1 DC-derived genes, but not others including ILC2s were parallelly altered, reflecting neutrophilic airway inflammation and AHR. Notably, *Fscn1*, the gene encoding fascin, was identified as one of the most significantly upregulated genes in cDC1s following ozone exposure in a TSLP-dependent manner (116). These data indicated that the TSLP signal via DC-derived molecules such as fascin might affect the mechanisms of non-type 2 airway inflammation in asthma, although further detailed experiments might be needed. Interestingly, in patients with chronic obstructive pulmonary disease (COPD), a representative of neutrophilic airway inflammation with similar airway obstructive disease as asthma, TSLP and IL-17A were expressed in induced sputum, and levels were higher than in healthy controls and healthy smokers (117). Recently, NET formation was found to be one of the mechanisms of induction of neutrophilic inflammation in various organs (118), as mentioned before, and in patients with psoriasis, TSLP promoted NET formation, and TSLP-primed NETs further amplified inflammatory cytokine production and oxidative stress, suggesting that the TSLP–NET axis has a pathogenic role (119). According to these data, TSLP has potential to mediate non-type 2 inflammation in patients with asthma.

### Osteopontin

Osteopontin (OPN), also known as secreted phosphoprotein-1 (*SPP1*), is a multifunctional phosphoglycoprotein involved in immune regulation, extracellular matrix remodeling, cell migration, and tissue repair, which as demonstrated in both human and animal studies (120). OPN interacts with integrins and CD44 receptors to regulate leukocyte trafficking and inflammatory responses (121). Several clinical studies have implied that OPN has an impact on asthma pathogenesis, including on severity, airway remodeling, inflammation, and AHR. Samitas et al. demonstrated increased OPN expression in bronchial biopsies from asthmatic patients, with levels correlating with disease severity and reticular basement membrane thickness, suggesting a role in airway remodeling on the human study (122). Increased OPN concentrations have also been detected in serum, sputum, and BALF of patients with asthma compared with healthy controls (122–124). In addition, increased OPN expression in the airways was correlated with impaired pulmonary function, a hallmark feature of severe asthma (125). Experimental models further support a pathogenic role for OPN. In OVA-induced allergic airway disease, OPN promotes eosinophilic inflammation, goblet cell hyperplasia, airway remodeling, and AHR in the animal studies (126, 127). These observations established OPN as a mediator of type 2-driven airway pathology. However, accumulating evidence suggests that OPN may also participate in non-type 2 inflammatory responses. Recent transcriptomic and mechanistic studies have broadened our understanding of OPN biology in the lung. OPN expression is increased in several neutrophilic pulmonary disorders, including COPD, acute lung injury, and viral respiratory infections (128–130). OPN can enhance neutrophil survival, migration, and activation through CD44- and integrin-dependent signaling pathways in the animal study (131), supporting a potential role in neutrophilic asthma. A recent study from our groups demonstrated that ozone exposure, a trigger of non-type 2 asthma, as mentioned above, markedly increased pulmonary OPN expression and neutrophilic airway inflammation with AHR. Using single-cell RNA sequencing, the authors identified monocyte-derived DCs (moDCs) as the predominant source of OPN in ozone-exposed lungs. CellChat analysis as a platform of cell-cell communication identification suggested active communication between moDC-derived OPN and neutrophils, macrophages, fibroblasts, pericytes, and airway structural cells. Functional experiments showed that reduction of moDC-associated populations reduced OPN production and attenuated ozone-induced airway inflammation and AHR, whereas OPN neutralization significantly suppressed neutrophilic airway inflammation. These findings establish a novel moDC–OPN–neutrophil axis in non-type 2 airway disease in the animal study (132). Collectively, the current evidence indicates that OPN is not merely a marker of airway remodeling, but also an active regulator of innate immune responses. The emerging role of OPN in pollutant-induced neutrophilic inflammation suggests that it may represent a promising biomarker and therapeutic target for severe non-type 2 asthma, a disease endotype for which effective targeted therapies remain limited.

### Gut microbiome

The gut microbiome has a huge impact on human health and modulates various disease conditions (133). Importantly, the gut microbiome affects obesity itself and obesity-induced diseases such as diabetes mellitus, cardiovascular diseases, and metabolic syndrome, and even augmentation of asthma, a major form of non-type 2 inflammatory phenotype in asthma (134). Accumulating evidence indicates that the gut microbiome plays a pivotal role in shaping immune responses beyond the gastrointestinal tract and contributes to the pathogenesis of asthma through the so-called gut-lung axis (134). Early-life microbial maturation appears particularly important, since delayed development of gut microbial diversity has been associated with an increased risk of childhood asthma in the human studies (135, 136). Alterations in the abundance of specific bacterial taxa, including *Bifidobacterium*, *Bacteroides*, *Faecalibacterium*, and *Roseburia* species, have been linked to asthma susceptibility and disease severity in the human studies (137, 138). A key mechanism underlying the gut-lung interaction involves microbial metabolites, especially short-chain fatty acids (SCFAs) such as acetate, propionate, and butyrate. These metabolites are generated through bacterial fermentation of dietary fiber and can affect systemic immune responses by modulating DC function, regulatory T-cell differentiation, and cytokine production (139, 140). Reduced fecal butyrate concentrations and decreased abundance of butyrate-producing bacteria have been reported in children with asthma, suggesting a protective role of SCFAs against airway inflammation in the human study (141). The gut microbiome may be particularly relevant in non-type 2 and obesity-associated asthma. Obesity is characterized by microbial dysbiosis, reduced microbial diversity, and altered metabolic outputs that promote systemic inflammation (142, 143). Recent studies have demonstrated distinct microbial signatures in obesity-related asthma, including reduced abundance of *Akkermansia muciniphila* and enrichment of several pro-inflammatory taxa in the human and animal model (144). Experimental models further support a causal role of the microbiome, showing that transfer of obesity-associated microbial communities enhances AHR and neutrophilic airway inflammation (38). In terms of the therapeutic candidates targeting the gut microbiome in asthma with non-type 2 inflammatory phenotypes, prebiotics and prudent use of antibiotics might be considered (26). In mice, ozone-induced AHR and neutrophilic airway inflammation augmented by obesity were attenuated by dietary fiber-enriched pectin, a fermentable fiber with significant manipulation of the gut microbiome component (38). In addition, macrolide antibiotics have attracted attention because, in addition to their antimicrobial activity, they exert immunomodulatory effects and can alter gut microbial composition. Clinical studies have demonstrated that long-term azithromycin therapy reduces asthma exacerbations in severe asthma (145), whereas experimental models of obesity-associated asthma suggested that macrolides may attenuate neutrophilic airway inflammation through modulation of microbiome-related inflammatory pathways and IL-17A signaling (37). Notably, there is still no conclusive evidence demonstrating that macrolide-induced modulation of the airway or gut microbiome directly reduces asthma exacerbations. Furthermore, direct evidence supporting the contribution of the immunomodulatory effects of macrolides to improvements in asthma pathophysiology remains limited. Hence, ongoing interest has focused on the possibility that azithromycin may improve outcomes in obesity-related severe asthma through modulation of the gut microbiome and systemic inflammatory pathways. Future studies should evaluate its effects on exacerbation rates and biomarker profiles, including cytokines and chemokines (146). Collectively, these findings suggest that microbiome-mediated immune and metabolic pathways contribute to non-type 2 inflammatory mechanisms in asthma and may represent promising therapeutic targets in future precision medicine approaches supported by the human and animal studies. Notably, the airway microbiome has also emerged as a potential contributor to non-type 2 asthma (147). Airway dysbiosis in severe asthma has been associated with poor asthma control, increased airway inflammatory cell burden, and enrichment of *Proteobacteria*, which correlates with Th17-related epithelial gene expression but not Th2-related gene signatures (148). These findings support a link between airway microbial dysbiosis and non-type 2 inflammation. Further studies are needed to determine whether modulation of the airway microbiome could provide therapeutic benefit in non-type 2 asthma.

### IL-6

IL-6 is considered an inflammatory cytokine reflecting systemic inflammation characterized by obesity (149), and, importantly, it might be a therapeutic target for patients with asthma (26). Indeed, in a U.S. cohort of patients with severe asthma, elevated plasma IL-6 levels were associated with more frequent asthma exacerbations and ED visits. IL-6 concentrations were positively correlated with BMI, and high IL-6 levels were observed more often in obese patients (BMI > 30 kg/m²; 38%) than in non-obese patients (BMI < 30 kg/m²; 8%), suggesting a role for IL-6 in obesity-related asthma severity in the human study (150). As mentioned previously, our data indicated that asthmatic patients with obesity showed higher exacerbation frequencies and lower pulmonary function with lower blood eosinophils than those without obesity. Interestingly, on serum cytokine and chemokine analyses, the rate of a high IL-6 level was greater in asthmatic patients with obesity than in those without obesity (32), showing that IL-6 might be considered a mechanistic molecule reflecting obesity-induced non-type 2 inflammatory phenotypes in the human study. In WT mice, ozone exposure increases neutrophils and IL-6 concentrations in BALF, and IL-6-deficient mice show significant reductions of neutrophils and granulocyte colony-stimulating factor (G-CSF) and IL-17A in BALF (151), indicating that G-CSF and IL-17A might be downstream molecules in IL-6-associated neutrophilic signaling in the animal study. In addition, ozone-induced elevation of IL-6 levels in BALF is greater in obese mice than in lean mice with different gut microbiomes (152), and germ-free mice reconstituted with obese mice-derived gut microbiomes showed higher IL-6 concentrations in BALF than germ-free mice reconstituted with lean mice-derived gut microbiomes (38), which indicates the possible effects of the gut microbiome and IL-6 on non-type 2 airway inflammation in asthma with obesity in the mice model. Clinically, IL-6 targeted biologics are available, but their efficacy for patients with asthma, especially the phenotypes of non-type 2 airway inflammation, is still controversial due to the limited availability of clinical trials with large sample sizes (153). According to these data, IL-6 might be a possible mechanistic molecule and therapeutic target for asthmatic mice with non-type 2 inflammatory phenotypes supported by several lines of basic research, but we still need clinical data on asthmatic patients with precise inflammatory phenotypes, even though we still have the issue of lacking biomarkers reflecting non-type 2 airway inflammation.

## Conclusion

**Figure 1:**
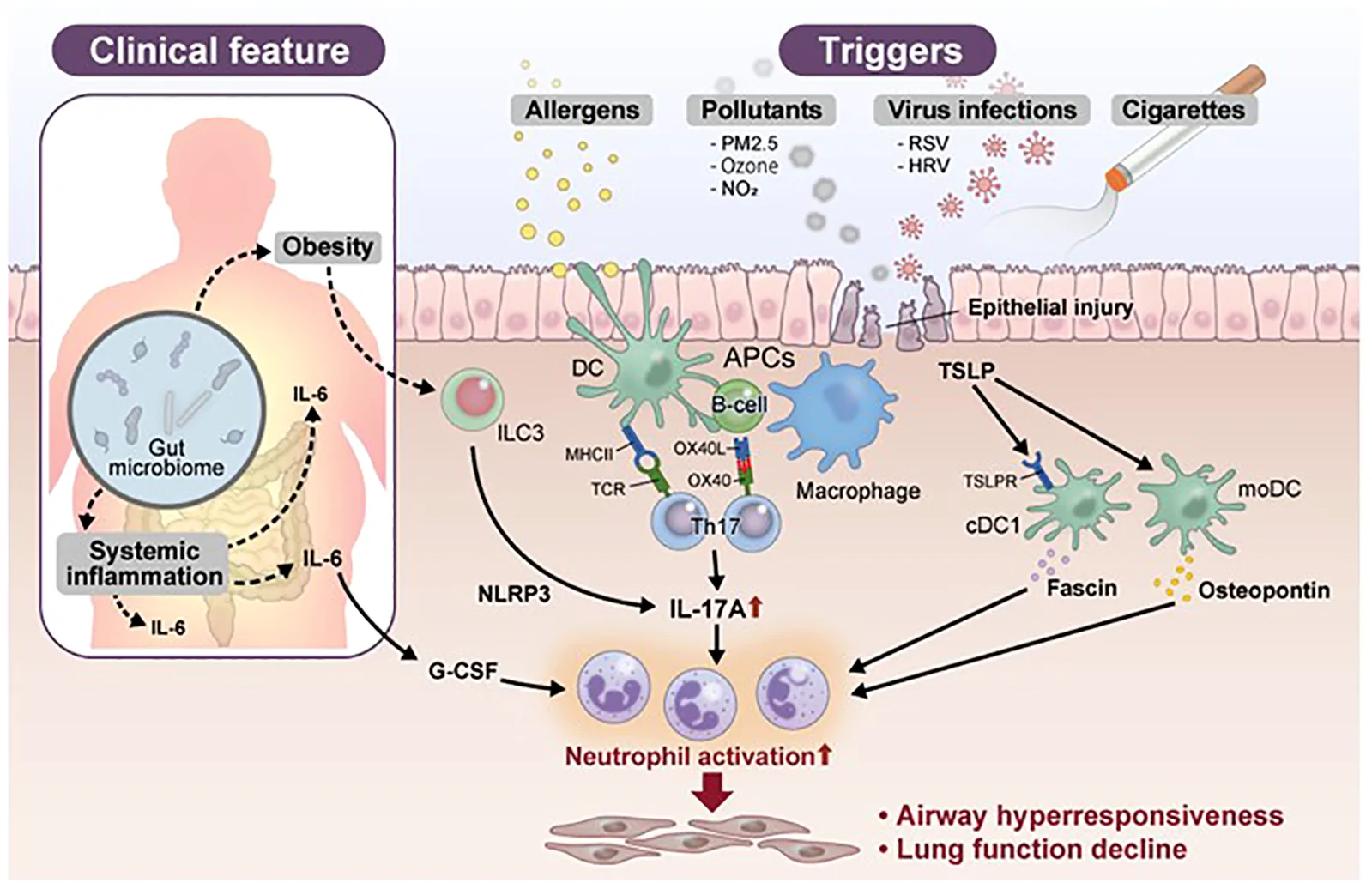
Proposed mechanisms underlying non-type 2 airway inflammation in severe asthma. Clinical features such as obesity and altered gut microbiome composition, together with external triggers including allergens, air pollutants, viral infections, and cigarette smoke, contribute to airway epithelial injury and the activation of innate and adaptive immune pathways. Obesity-associated systemic inflammation promotes IL-6 production, which may enhance G-CSF- and IL-17A-associated neutrophilic responses. The gut-lung axis and NLRP3 inflammasome activation may further promote ILC3- and Th17-mediated IL-17A production. Antigen-presenting cells, including dendritic cells, macrophages, and B cells, promote Th17 differentiation and activation through the MHCII–TCR interaction and costimulatory pathways such as OX40L–OX40. Airway epithelial injury induced by pollutants, viral infections, or cigarette smoke leads to the release of TSLP, which activates TSLPR-expressing cDC1s and promotes molecules such as fascin. In parallel, monocyte-derived dendritic cells produce osteopontin, which contributes to neutrophil recruitment and activation. These converging pathways increase IL-17A production and neutrophilic airway inflammation, resulting in airway hyperresponsiveness and progressive lung function decline. APCs, antigen-presenting cells; cDC1, conventional dendritic cell type 1; DC, dendritic cell; G-CSF, granulocyte colony-stimulating factor; HRV, human rhinovirus; IL, interleukin; ILC3, group 3 innate lymphoid cell; Mac, macrophage; MHCII, major histocompatibility complex class II; moDC, monocyte-derived dendritic cell; NLRP3, NLR family pyrin domain-containing 3; NO_2_, nitrogen dioxide; OX40L, OX40 ligand; PM2.5, particulate matter with an aerodynamic diameter ≤2.5 μm; RSV, respiratory syncytial virus; TCR, T-cell receptor; Th17, T helper 17 cell; TSLP, thymic stromal lymphopoietin.

The population of severe asthma patients with non-type 2 inflammation is heterogeneous and is characterized by neutrophilic airway inflammation, corticosteroid insensitivity, and limited responsiveness to currently available type 2-targeted biologics. Clinical and experimental evidence suggests that obesity, air pollution, cigarette smoke, and respiratory viral infections contribute to disease development and exacerbation through activation of IL-17A-associated pathways and innate immune responses. Recent studies have identified several potential therapeutic targets, including IL-17A, TSLP, OPN, the gut microbiome, and IL-6, highlighting the complex interplay of environmental factors, metabolism, and immune regulation in severe asthma with non-type 2 airway inflammation (**Figure 1**). However, effective targeted therapies for these phenotypes remain limited, and reliable biomarkers for patient stratification are still lacking. Further integration of clinical phenotyping with mechanistic and translational research will be essential to better define non-type 2 asthma endotypes and to develop precision medicine approaches that improve outcomes in patients with severe asthma.
